# The MicroRNA Interaction Network of Lipid Diseases

**DOI:** 10.3389/fgene.2017.00116

**Published:** 2017-09-22

**Authors:** Abdul H. Kandhro, Watshara Shoombuatong, Chanin Nantasenamat, Virapong Prachayasittikul, Pornlada Nuchnoi

**Affiliations:** ^1^Center for Research and Innovation, Faculty of Medical Technology, Mahidol University Bangkok, Thailand; ^2^Center of Data Mining and Biomedical Informatics, Faculty of Medical Technology, Mahidol University Bangkok, Thailand; ^3^Department of Clinical Microbiology and Applied Technology, Faculty of Medical Technology, Mahidol University Bangkok, Thailand; ^4^Department of Clinical Microscopy, Faculty of Medical Technology, Mahidol University Bangkok, Thailand

**Keywords:** microRNA, text mining, interaction network, lipid diseases, dyslipidemia

## Abstract

**Background:** Dyslipidemia is one of the major forms of lipid disorder, characterized by increased triglycerides (TGs), increased low-density lipoprotein-cholesterol (LDL-C), and decreased high-density lipoprotein-cholesterol (HDL-C) levels in blood. Recently, MicroRNAs (miRNAs) have been reported to involve in various biological processes; their potential usage being a biomarkers and in diagnosis of various diseases. Computational approaches including text mining have been used recently to analyze abstracts from the public databases to observe the relationships/associations between the biological molecules, miRNAs, and disease phenotypes.

**Materials and Methods:** In the present study, significance of text mined extracted pair associations (miRNA-lipid disease) were estimated by one-sided Fisher's exact test. The top 20 significant miRNA-disease associations were visualized on Cytoscape. The CyTargetLinker plug-in tool on Cytoscape was used to extend the network and predicts new miRNA target genes. The Biological Networks Gene Ontology (BiNGO) plug-in tool on Cytoscape was used to retrieve gene ontology (GO) annotations for the targeted genes.

**Results:** We retrieved 227 miRNA-lipid disease associations including 148 miRNAs. The top 20 significant miRNAs analysis on CyTargetLinker provides defined, predicted and validated gene targets, further targeted genes analyzed by BiNGO showed targeted genes were significantly associated with lipid, cholesterol, apolipoprotein, and fatty acids GO terms.

**Conclusion:** We are the first to provide a reliable miRNA-lipid disease association network based on text mining. This could help future experimental studies that aim to validate predicted gene targets.

## Introduction

Dyslipidemia is a common form of lipid disorder; characterized by increased levels of TGs, increased LDL-C, and decreased level of HDL-C. The low levels of HDL-C and high levels of LD-C are the most imperative factors for the development of cardiovascular disease (CVD), especially ischemic heart disease and stroke (Meagher, 2004). The liver is the major organ where cholesterol, lipid, and lipoprotein synthesis and metabolism taking place (Min et al., 2012). Recently, miRNAs have been reported to modulate these processes (Esau et al., 2006; Moore et al., 2010; Vickers et al., 2013). The miRNA-122 is identified for the involvement of the regulation of lipid metabolism (Esau et al., 2006). A recent study that shows miRNA-27b targeted to 27 of 151 lipid-associated genes. This therefore indicates that miRNA-27b serves as a key molecule for post-transcriptional hub of lipid metabolism genes. In mice model, *GPAM* is one of the key lipid metabolism gene targeted by miR-27b. The up-regulation of hepatic miR-27b is associated with decrease *GPAM* mRNA and plasma triglyceride (Vickers et al., 2013).

Moreover, miRNA-33a and miRNA-33b have been extensively identified to be involved in cholesterol and lipid homeostasis. miRNA33a and miRNA-33b are experimentally characterized to be located in intronic regions of *sterol regulatory elementary binding protein-2 and 1* (*SREBP2, SREBP1*), respectively (Marquart et al., 2010; Najafi-Shoushtari et al., 2010; Rayner et al., 2010). The finding of hepatic miR-27b is promising for modulating the endogenous miR-27b level as effective therapeutic approach in lipid related disorder in further study.

miRNAs are evolutionary conserved and found commonly in humans, flies, plants, and viruses (Lagos-Quintana et al., 2003). Signaling proteins, metabolic enzymes, transcription factors are regulated by miRNAs. The expression levels of miRNAs have demonstrated their potential usage as biomarkers for various diseases (Lagos-Quintana et al., 2003). Although there have been advancements in miRNA profiling, the experimental process for searching disease related miRNAs is considerably expensive and time-consuming (Jiang et al., 2010, 2013). Computational approaches have been proposed to overcome these limitations and drawbacks in miRNA research. A large number of miRNA prediction softwares have been developed to predict miRNA by targeting 3′UTR of mRNA such as, miRanda (Enright et al., 2004), TargetScan (Lewis et al., 2003), and PicTar (Krek et al., 2005). The computational prediction algorithms mostly analyze the binding of miRNA at 3′UTR region of human mRNA. With the public availability of human genome information and miRNA bioinformatics tools, a large number of research publications related to miRNA in human diseases have been published, and are now in databases such as, PubMed and Scopus. Text-mining is one of the promising tools for depicting the body of knowledge from the literature. Naeem et al. (2010) demonstrated the use of co-occurring based text mining method for elucidating miRNA-gene association. Murray et al. (2010) uncovered human miRNA-target interactome (microRNAome), using natural language processing (NLP) based text-mining, network analysis, and ontological enrichment methods. Goh et al. (2007) described the involvement of 176 miRNAs and their target genes in the controlling of 368 OMIM disorders using human disease network. There is a need for data reduction methods i.e., text-mining, that utilize validated miRNA-disease associations from experimental published abstracts which indicates most significantly disease related miRNAs for experimental study.

We constructed a miRNA-lipid disease association network using computational approaches; including text-mining approach with miRNA bioinformatics tools. This is the first study that delineates the interacting network of miRNAs and the target genes in human lipid disorders.

## Materials and methods

### Data collection

**Figure 1:**
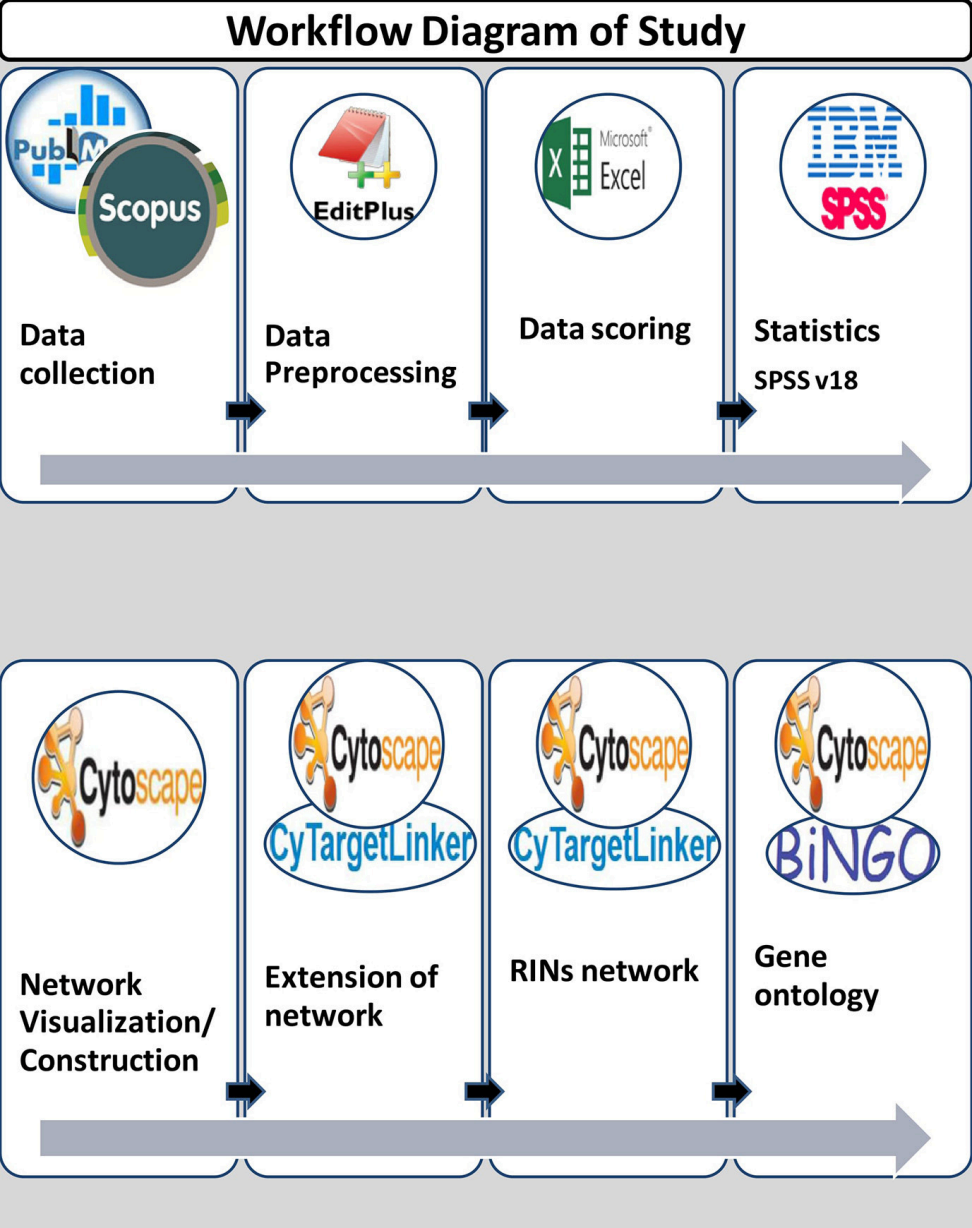
Workflow diagram of study.

In this study, data was collected from January 1, 2000 to December 31, 2013. A total of 730 abstracts were collected from publicly available databases like PubMed and Scopus by using keywords for lipid diseases/identifier and miRNA terms. Figure 1 shows the workflow diagram of the present study.

In the present study, the text mining framework was divided into five main steps, namely:

- Information Retrieval (IR)
- Information Extraction (IE) & Scoring
- MicroRNA-disease Network Construction
- Extension of network to predict new miRNA targets
- Identification of Gene Ontology (GO) terms for predicted targets.

**Figure 2:**
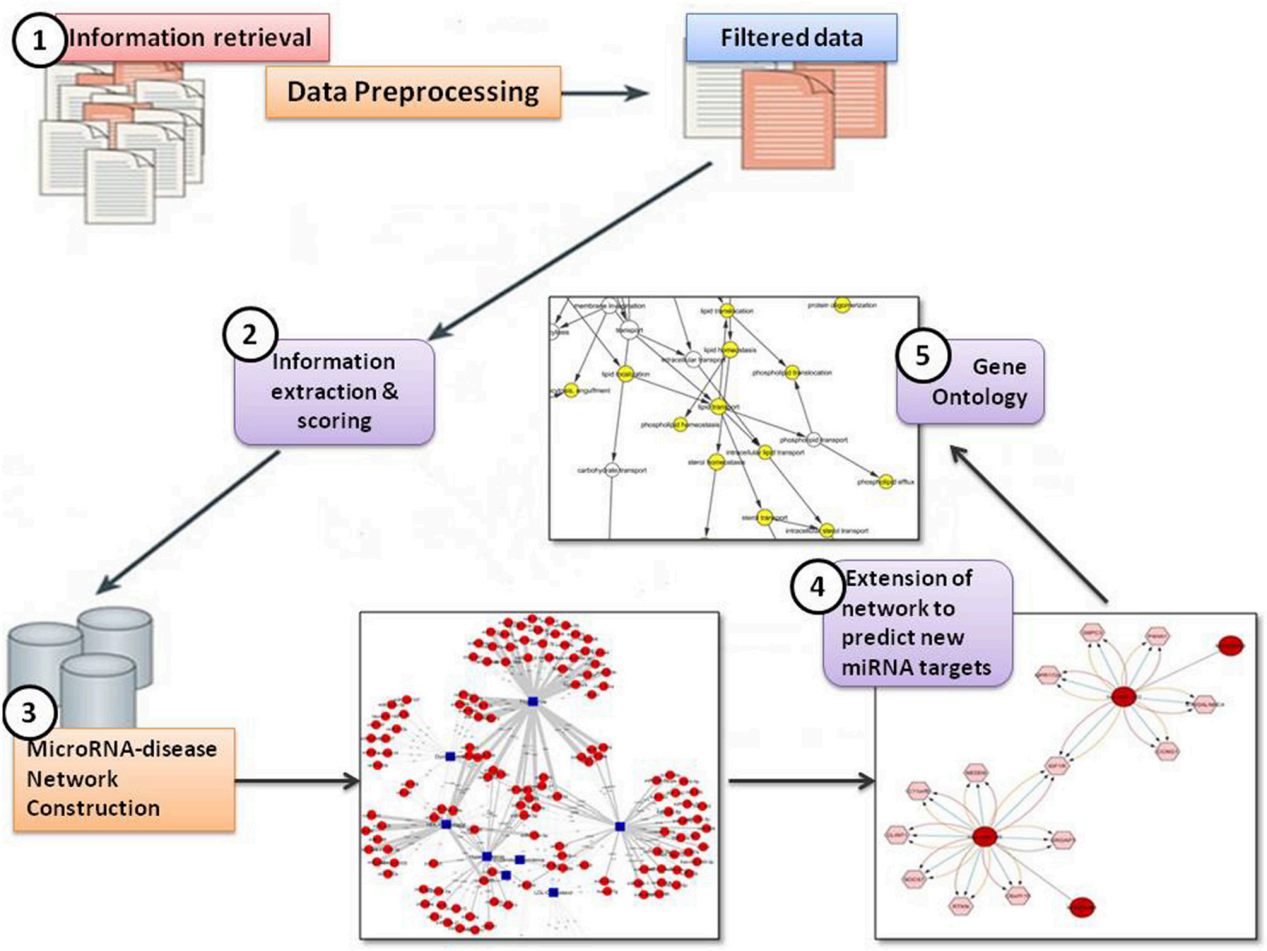
Text-mining framework.

Figure 2 shows the text-mining framework into five steps

### (i) Information retrieval (IR)

The fundamental assumption in the field of text mining is that co-occurrence means association. Based on the co-occurrence assumption, the associations between different miRNAs and lipid diseases or identifiers were determined. If a particular miRNA and lipid disease or identifier were mentioned in the same abstract, we assumed that they co-occurred and were associated.

Information retrieval highly relies on the keyword recognition, which is the miRNA name and disease or disease identifier groups, then the set of keywords used to search within the databases and retrieve the keywords containing abstracts. We used different keyword terms for miRNA prefixes, because of the different patterns of recognition for their names such as: “MicroRNA,” “MiRNA,” “miR,” prefixed species as “hsa-miR-1,” as precursor “pre-miR-1,” as loci or variant “miR-1a-1.” Other variants like “lin-4” and “let-7,” as an abbreviation more than one miRNA “miR-221/222” and “miR-15 & –16,” However, for disease or identifier groups, we selected the four common lipid diseases and seven identifiers which are related to the 4 common diseases. The common diseases are Dyslipidemia, Hyperlipidemia, Hypercholesterolemia, and Hypertriglyceridemia, while the 7 Identifiers such as, HDL-Cholesterol, HDL, LDL-Cholesterol, LDL, Triglyceride, Low HDL-C, and High LDL-C, Low HDL-C. We made a pair of miRNA+disease or miRNA+identifiers for searching abstracts from both PubMed and Scopus databases from January 1, 2000 to December 31, 2013, and saved these as a notepad file (for example: “MicroRNA and Dyslipidemia,” “MiRNA and Dyslipidemia,” “miR and dyslipidemia,” “hsa-miR and Dyslipidemia,” and similarly other miRNA recognition terms).

### (ii) Information extraction (IE) and scoring

The simplest form of the approach used in our study depend on the relation of keywords between abstracts is an association based on the co-occurrence of the keywords in the text. When two keywords are frequently mentioned in the abstract, an association relation between keywords is inferred. By using EditPlus software (https://www.editplus.com/), which is used for manual text mining or information extraction (IE) from reference abstracts.

The significance level of extracted miRNA-disease association pairs were computed by one-sided Fisher's exact tests (Fisher, 1922). The *P*-value of Fisher's exact tests (Fisher, 1922) was calculated based on hypergeometric distribution, as follows:

P=(a+b)!(c+d)!(a+c)!(c+d)!/(a!b!c!d!n!)

where ***n*** is denoted the total number of abstracts included in text mining;

**a** is the ***True positive (TP)*** which represents the number of abstracts that contain both the miRNA and disease;
**b** is the ***False positive (FP)*** which represents the number of abstracts that contain only miRNA;
**c** is the ***False negative (FN)*** which represents the number of abstracts that contain only the disease/identifier;
and **d** is the ***True negative (TN)*** which represents the number of abstracts that don't contain either terms.

The *P*-value, which determines whether a miRNA and disease have a link, is considered significant as ≤**0.05**.

### (iii) MicroRNA-disease network construction

One of the commonly used framework to visualize and analyze biological network is Cytoscape (Shannon et al., 2003). It provides functionality for representation and integration of biomolecular network models. In present study, we constructed the bipartite network by mapping pairs of miRNA-disease associations based on *P*-values, and visualized the network by Cytoscape v3.2.0. Here, the disease groups attributed to a node, and miRNAs attributes to an edge. The interaction between disease and miRNA weighted with corresponding *P*-values. Each edge in the network connects a miRNA and one of its corresponding one or more than one disease group, similarly each disease group corresponds one miRNA or more than one miRNAs. Thus, resulting constructed miRNA-disease association network provides information on whether miRNA is associated with a disease.

### (iv) Extension of network (new miRNA targets predictions)

Cytoscape has a modular structure and extension of networks with additional functionalities is possible through apps (formerly known as plugins). Currently, few Cytoscape apps are available that either extend networks with other types of molecular interaction data or focus on one specific type of regulatory interaction. A new Cytoscape app, CyTargetLinker (Kutmon et al., 2013) allows users to build regulatory interaction networks, and allow their inclusion in the network analysis process.

**Figure 3:**
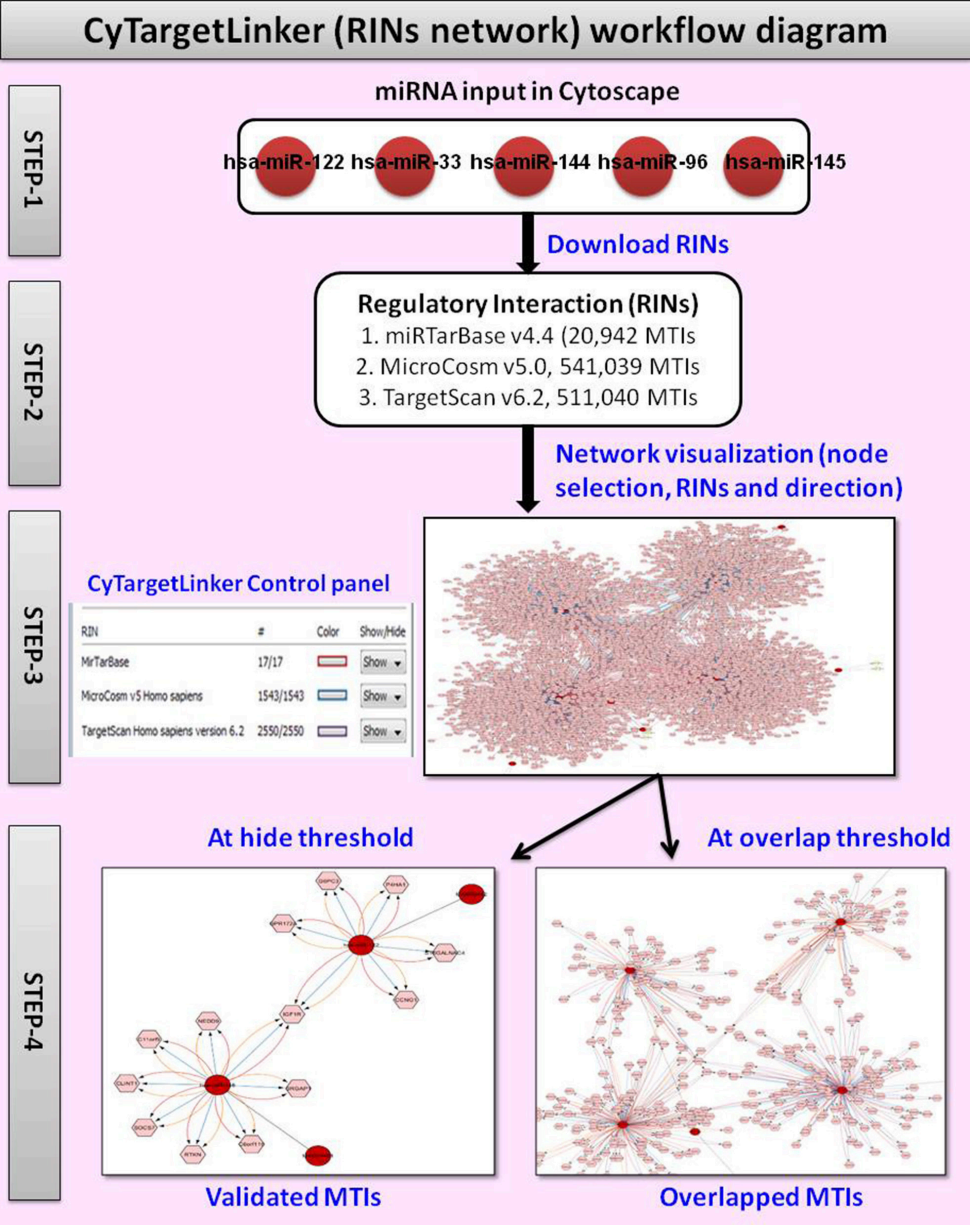
Workflow diagram of CyTargetLinker regulatory interaction network.

We used CyTargetLinker v3.0.1 to validate and predict miRNA target interactions (MTIs) and visualize them in a graphical way by extension of the network. A regulatory interaction network (RegIN) is a network containing regulatory interactions often derived from online interaction databases. To construct a RegIN with CyTargetLinker on Cytoscape, we obtained Homo sapiens MTIs from one experimentally validated database miRTarBase v4.4, which includes 20,942 MTIs, and from two predicted miRNA databases; MicroCosm v5.0, which includes 541,039 MTIs and TargetScan v6.2, which includes 511,040 MTIs. The networks are stored in XGMML (the eXtensible Graph Markup and Modeling Language) format, which is supported by Cytoscape. Each regulatory interaction consists of two nodes, a source (regulatory component) and target biomolecule, connected through one directed edge. The CyTargetLinker website http://projects.bigcat.unimaas.nl/cytargetlinker/regins provides a collection of RegINs for different species and interaction types. Figure 3 shows the workflow diagram of CyTargetLinker.

In present study, top 20 significant miRNAs selected for extension of network by CyTargetLinker. The top 20 significant miRNAs then divided into four sets (each set contains five miRNAs) with miRBase accession numbers and used as input file for CyTargetLinker.

➢ The first step is to load input file on Cytoscape.
➢ In the second step, Cytoscape visualize as grid layout, then we select perfused force directed layout and launch CyTargetLinker from application manager to integrate MTIs.
➢ In the third step the CyTargetLinker integration and extension of the network process is started. In the dialogue box before network extension user can add either targets or regulators or both as default. As a result CyTargetLinker extracts the RINs from the provided MTIs. After the extension of network, CyTargetLinker fix different colors on each edge for targets, regulators, and MTIs. We can see the detail on control panel, where color selection and number of MTIs for each databases is listed.
➢ In the fourth step the network MTIs can be visualized by adopting the *hide/show* and/or *overlap threshold* function. The function of *hide/show* key is enables the temporary removal of specific MTIs and showing only the interactions from a subset of loaded MTIs. However, the function of *overlap threshold* key is to show only the interactions that are supported by a defined number of MTIs or more. After the most targeted MTIs visualized in Cytoscape/CyTargetLinker RIN network, the targeted MTIs were used to retrieve the GO for identifying their biological processes. For GO, another Cytoscape Plug in tool, BiNGO (Maere et al., 2005) was used.

### (v) Gene ontology

**Figure 4:**
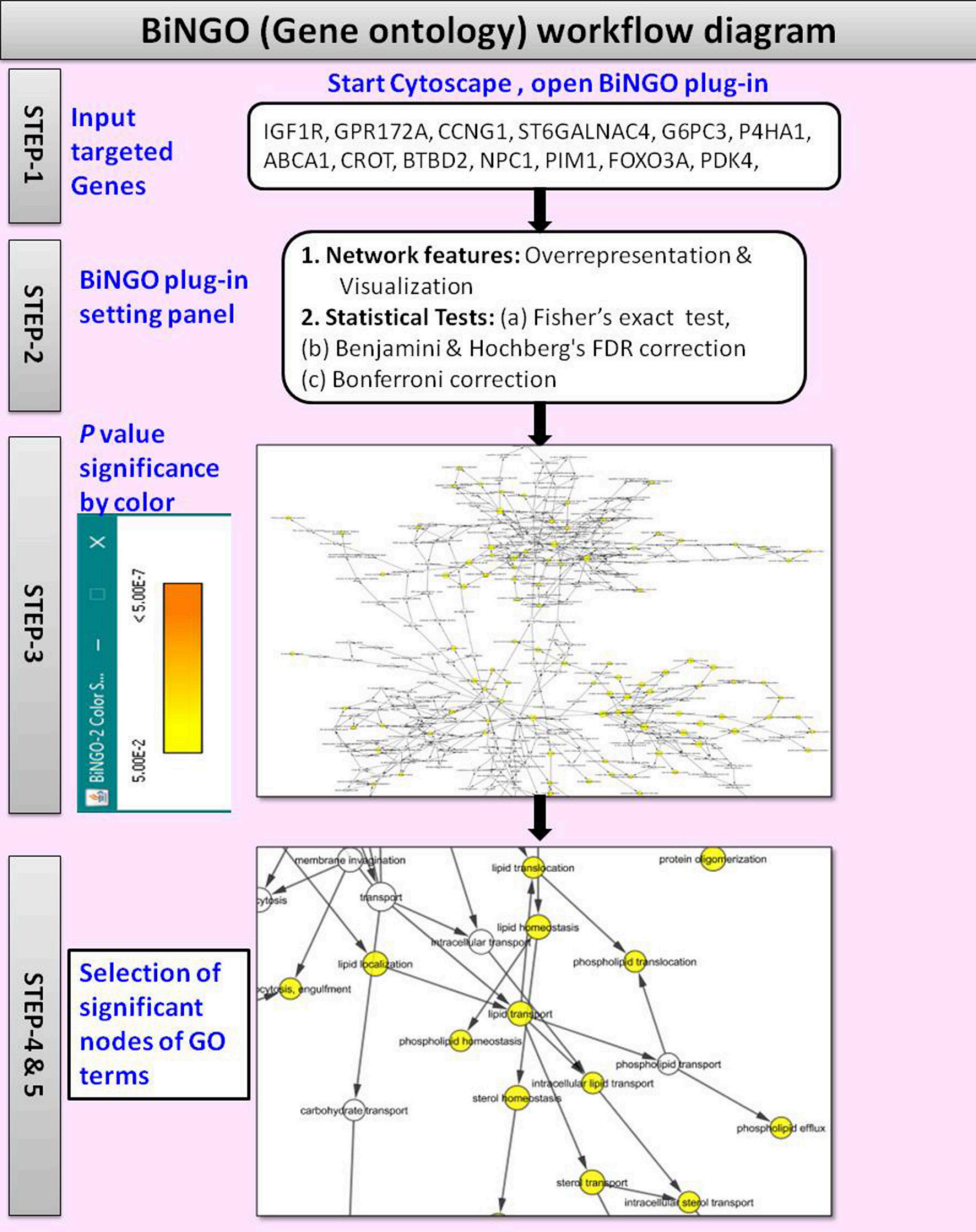
Workflow diagram of BiNGO Gene ontology.

The BiNGO v3.0.3 (Maere et al., 2005) is a Cytoscape plugin tool used to retrieve the GO annotations for the targeted genes identified with CyTargetLinker. Figure 4 shows the workflow diagram of BiNGO Gene ontology analysis.

By using the input list of targeted genes; BiNGO accesses the overrepresentation of GO categories in a subgraph of a biological network, which is visualized on Cytoscape. The enrichment of GO terms in the targeted genes was evaluated with a right-sided hypergeometric statistical analysis. The hypergeometric test *P*-value was set to ≤0.05, and the Benjamini and Hochberg correction was applied to provide strong control over the false discovery rate under positive regression dependency of the test statistics. After statistical analysis, the GO hierarchy was visualized as overrepresented GO categories.

The main advantages of BiNGO are:

- It supports GOSlim ontologies (Consortium, 2004),
- It offers enormous flexibility in the use of ontologies and annotations,
- It can be integrated with a range of molecular networks including protein-protein interactions or transcriptional co-regulation networks,
- It allows networks to be modified, viewed and analyzed in various ways on Cytoscape.

## Results

### MicroRNA-lipid disease association analysis

In the present study, the associations were identified by co-occurrence-based manual text-mining approach and significance was measured by one-sided Fisher's exact *P*-values. Significant associations were used to construct the network on Cytoscape. By processing 730 publications, we recorded 227 pairs of miRNA-lipid disease associations. Among these associations, there are 148 miRNAs and 09 (04 diseases, 05 identifiers) groups involved. Table 1 gives an overview of the number of miRNAs, diseases, miRNA- lipid disease associations, and a number of papers.

**Table 1:** Summary of the number of miRNAs, diseases/identifiers, miRNA-disease associations, and number of papers.

**Components**	**Fisher's exact**
Total No. of miRNAs	148
No. of disease/Identifier groups	9
miRNA-disease occurrence/Associations	227
No. of papers in pairs associations	313
Total No. of papers	414

### MicroRNA-lipid disease association network construction and visualization

**Figure 5:**
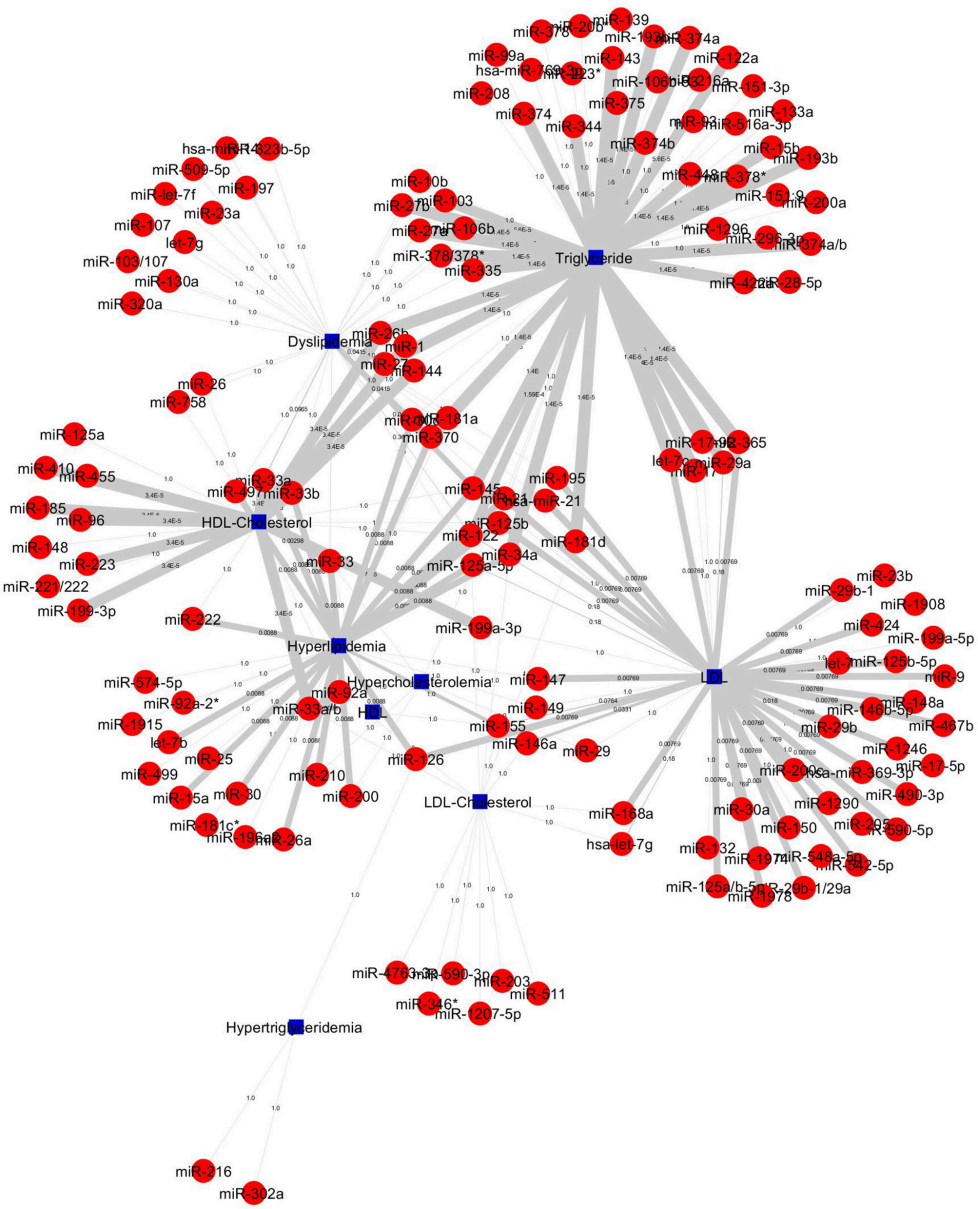
All 227 miRNA-lipid disease associations by *P*-values. Red circles and green circles represent miRNAs and diseases, blue square represents lipid diseases/identifiers, respectively, according to the number of corresponding text mined annotated papers. Each linked pair represents a miRNA-disease association with edge-weighted measurement by *P*-values to visualize the strength of the miRNA-disease association. The miRNAs either connect one disease or more than one disease; it shows that a single miRNA or group of miRNAs may be involved with one or more than one disease.

**Figure 6:**
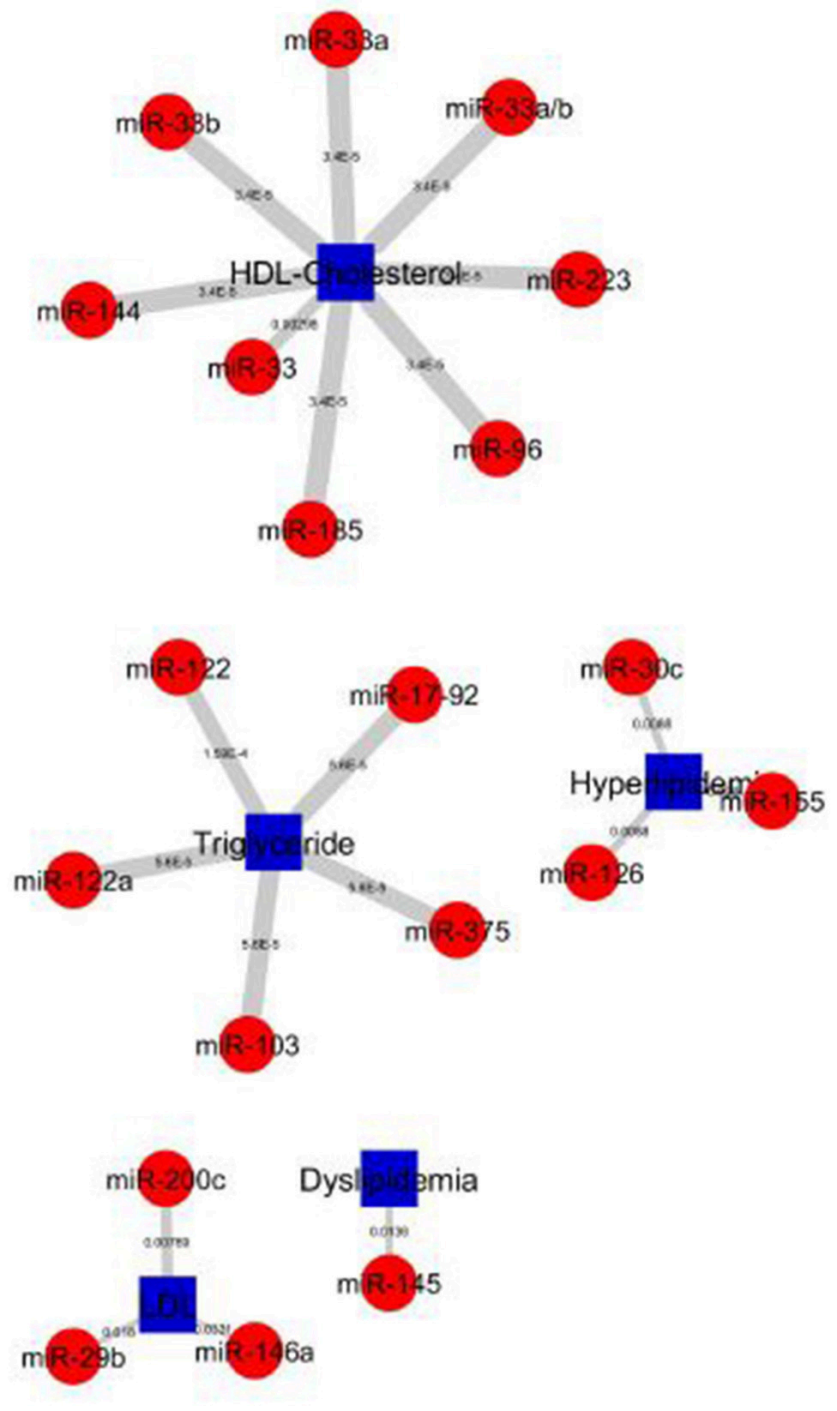
Top 20 miRNA-lipid disease association of *P*-values. Red circles and green circles represent miRNAs and diseases, blue square represents lipid diseases/identifiers, respectively, according to the number of corresponding, text-mined annotated papers. Each linked pair represents an miRNA-disease association with edge-weighted measurement by *P*-values to visualize the strength of the miRNA-disease association. The higher strength is shown in HDL-Cholesterol and Triglyceride groups paired with miRNAs.

The construction of bipartite network of miRNA-disease associations based on the *P*-values. The *P*-values of each association was computed by one-sided Fisher's exact test, and were calculated based on hypergeometric distribution. The bipartite network consists of 157 nodes (corresponding to disease/identifier and miRNAs) and 227 edges (corresponding to miRNA-disease associations). We prioritized 148 miRNAs in 4 diseases and 5 identifier groups and all miRNA-disease associations shown in Figure 5. The top 20 significant association network constructed which is based on edge-weighted *P*-values, shown in Figure 6. The higher significant *P*-values correspond to more thicker edges between each pair. The higher strength is shown in HDL-Cholesterol and Triglyceride group paired miRNAs.

The top 20 significant miRNAs-disease pairs selected with a number of papers by applying the one-sided Fisher's exact *P*-values. Table 2 shows the top 20 significant associations with *P*-values. From the nine disease/identifier groups, only four groups show the higher number of pairs including, HDL-Cholesterol, Triglyceride, Hyperlipidemia, LDL group as seen in Table 2. The miRNA-33 family have shows a higher number of papers and mostly paired in the HDL-Cholesterol group.

**Table 2:** Top 20 significant associations between miRNA and disease *P*-value by one-sided Fisher's exact method.

**S. No**.	**miRNAs**	**Disease group**	**No. of papers**	***P*-value**
1	miRNA-33a	HDL-Cholesterol	7	3.40E-05
2	miRNA-33a/b	HDL-Cholesterol	7	3.40E-05
3	miRNA-144	HDL-Cholesterol	3	3.40E-05
4	miRNA-223	HDL-Cholesterol	3	3.40E-05
5	miRNA-33b	HDL-Cholesterol	3	3.40E-05
6	miRNA-185	HDL-Cholesterol	2	3.40E-05
7	miRNA-96	HDL-Cholesterol	2	3.40E-05
8	miRNA-103	Triglyceride	2	5.60E-05
9	miRNA-122a	Triglyceride	2	5.60E-05
10	miRNA-17-92	Triglyceride	2	5.60E-05
11	miRNA-375	Triglyceride	2	5.60E-05
12	miRNA-122	Triglyceride	5	1.59E-04
13	miRNA-33	HDL-Cholesterol	16	2.98E-03
14	miRNA-200c	LDL	2	7.69E-03
15	miRNA-126	Hyperlipidemia	2	8.80E-03
16	miRNA-30c	Hyperlipidemia	2	8.80E-03
17	miRNA-145	Dyslipidemia	2	1.36E-02
18	miRNA-29b	LDL	3	1.80E-02
19	miRNA-155	Hyperlipidemia	2	2.80E-02
20	miRNA-146a	LDL	4	3.31E-02

### Construction of regulatory interaction network (new miRNA target predictions) by CyTargetLinker on cytoscape

The CyTargetLinker on Cytoscape used to extend the RIN network, which augments user knowledge about the new miRNA target predictions that could be used for further experimental studies. To get better insight from present top 20 miRNA target predictions and extend our RegIN, we used CyTargetLinker Plug in application on Cytoscape. We obtained experimentally validated MTIs by miRTarBase database and predicted MTIs by MicroCosm and TargetScan databases, which are described in Table 3, where the number of nodes, edges, number of validated, and predicted targets is listed as follows:

**Table 3:** CyTargetLinker's validated and predicted MTIs.

**Method**	**Nodes**	**Edges**	**miRTarBase (experimental validated MTIs)**	**MicroCosm (predicted MTIs)**	**TargetScan (predicted MTIs)**
Fisher's Set-1	4,356	5,399	361	2,851	2,174
Fisher's Set-2	4,082	4,941	94	2,756	2,078
Fisher's Set-3	6,064	8,361	1,043	4,353	2,951
Fisher's Set-4	4,889	6,295	727	3,315	2,242

**Figure 7:**
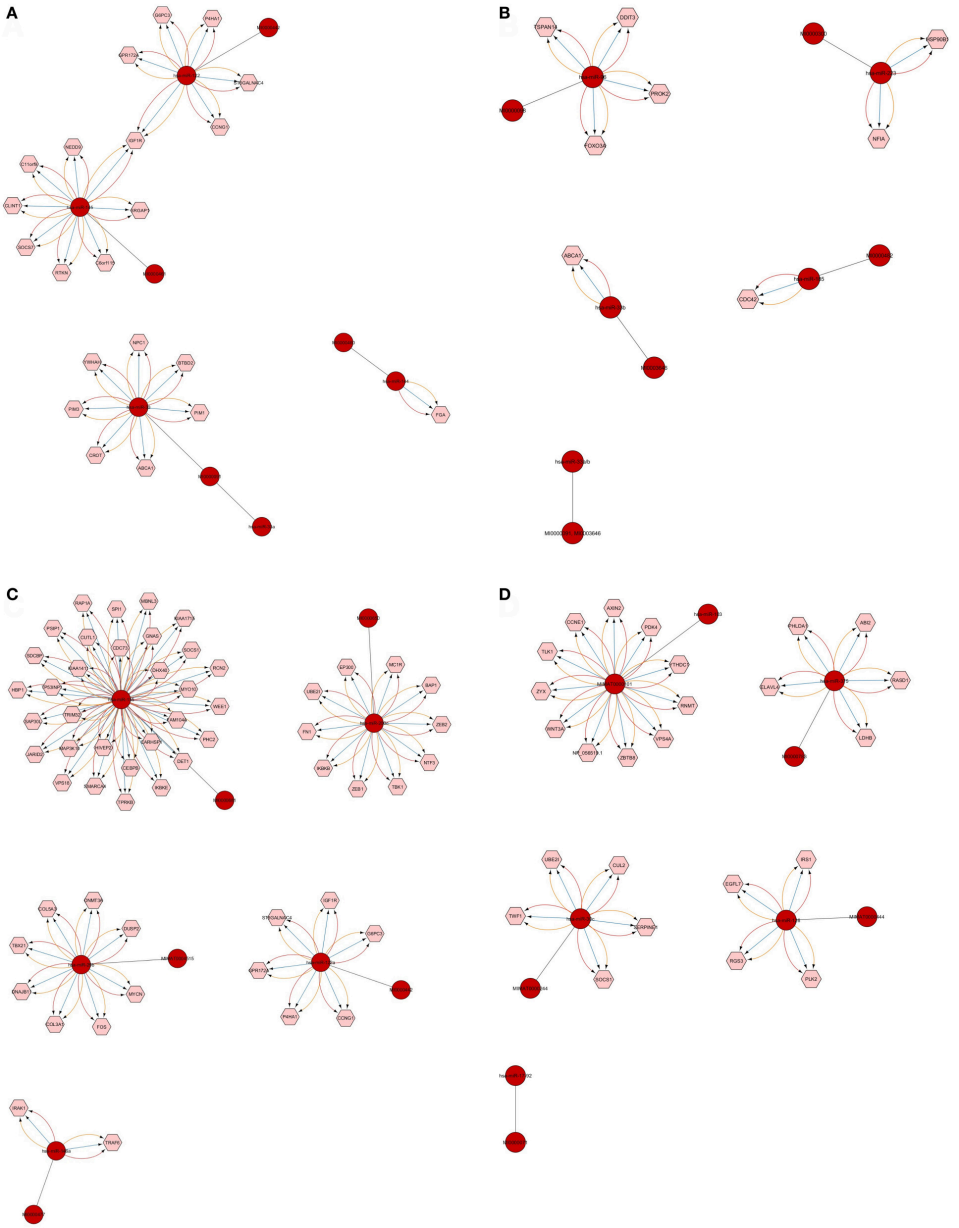
Defined number of predicted and validated target genes from Top 20 significant miRNAs. Among the 20 significant miRNAs, only 18 showed a defined number of targets at threshold 3 functionality modes on CyTargetLinker and Cytoscape. The miRNAs and their accession numbers are in red circles; pink hexagonal shapes indicate their regulatory interaction (targeted mRNAs/genes). The different colors of arrows show regulatory interactions for the identification based on miRNA validated and predicted databases (miRTarBase, MicroCosm, and TargetScan). Each miRNA targets mRNA/genes and the high number of such targets is shown in **(C)** where miRNA-155 has 31 targets and miRNA-200c has 11. In **(D)** miRNA-103 has 11 targets. Single targets also were found as shown in **(A)** for miRNA-144 and **(B)** for miRNA-33b and miRNA-185.

We found that CyTargetLinker provides quick and extensive enrichment of biological network with regulatory information. At threshold 3 functionality on the control panel, we observed the defined number of targeted genes that provide regulatory interactions of validated and predicted MTIs, and shown in Table 4 and Figure 7.

**Table 4:** CyTargetLinker results of defined number of targets by threshold functionality.

**Method and miRNA sets**	**MTIs by (miRTarBase, MicroCosm, TargetScan)**
Fisher's Set-1	22
Fisher's Set-2	8
Fisher's Set-3	57
Fisher's Set-4	25
Total	112

The number of defined targeted genes by miRNAs is shown in Figure 7. The higher to lower number of MTIs are; miRNA-155 targeted 31 genes, miRNA-103 targeted 11 genes, and miRNA-200c targeted 10 genes. The targeted genes are derived from validated and predicted MTIs databases. The defined numbers of each miRNA target genes are listed in Table 5.

**Table 5:** Top 20 significant miRNAs and their predicted and validated targets by CyTargetLinker extension network analysis.

**miRNAs**	**Targets**
hsa-miR-122	*IGF1R, GPR172A, CCNG1, G6PC3, P4HA1, ST6GALNAC4*
hsa-miR-145	*IGF1R, NEDD9, SOCS7, C6orf115, SRGAP1, CLINT1, RTKN, C11orf9*
hsa-miR-33, hsa-miR-33a	*YWHAH, PIM3, ABCA1, CROT,BTBD2,NPC1,PIM1*
hsa-miR-144	*FGA*
hsa-miR-96	*PROK2, TSPAN14, DDIT3, FOXO3A*
hsa-miR-223	*HSP90B1, NFIA*
hsa-miR-185	*CDC42*
hsa-miR-33b	*ABCA1*
hsa-miR-155	*SDCBP, VPS18, CUTL1, TRIM32, MYO10, SAP30L, RCN2, CEBPB, SOCS1, MBNL3, SMARCA4, PSIP1, TP53INP1, IKBKE, DET1, DHX40, MAP3K10, WEE1, HBP1, RAP1A, SPI1, GNAS, KIAA1715, KIAA1411, FAM104A, TPRKB, HIVEP2, CDC73, CARHSP1, JARID2, PHC2*
hsa-miR-200c	*BAP1, EP300, MC1R, ZEB1, IKBKB, NTF3, UBE2I, ZEB2, TBK1, FN1*
hsa-miR-122a	*CCNG1, G6PC3, ST6GALNAC4, IGF1R, P4HA1, GPR172A*
hsa-miR-29b	*DNMT3A,COL3A1,TBX21, COL5A3, DUSP2, FOS, MYCN, DNAJB11*
hsa-miR-146a	*TRAF6, IRAK1*
hsa-miR-103	*ZYX, YTHDC1, ZBTB8, PDK4, RNMT, CCNE1, VPS4A, TLK1, AXIN2 NP_056519.1, WNT3A*
hsa-miR-375	*ELAVL4, ABI2, LDHB, PHLDA1, RASD1*
hsa-miR-126	*IRS1, EGFL7, RGS3, PLK2*
hsa-miR-30c	*UBE2I, CUL2, SOCS1, SERPINE1, TWF1*

### Gene ontology analysis

Besides the RegIN information, next step to obtain biological functions of targeted genes for further understanding the biological role of the gene. To gain further insight into the molecular aspects of above listed miRNAs signature in lipid disorders, we investigated the GO for biological, cellular, and molecular processes associated with a set of predicted and validated targeted genes by miRNAs. Surprisingly, we found more than 90 related GO terms shown in Supplementary Material among which more than 20 GO terms were significantly associated with lipids, cholesterol, fatty acid, apolipoproteins, sterol, and insulin and shown in Table 6. When we narrowed down searching on molecular and cellular processes related GO terms, at this stage the GO terms were associated with gene activity, negative and positive regulation of metabolic process, regulation of biological process, metabolic processes, cellular metabolic processes, transportation of lipids, storage of lipids, cholesterol efflux, and macromolecular biosynthetic processes.

**Table 6:** Top significant related gene ontology terms of CyTargetLinker targeted genes analyzed by BINGO on Cytoscape.

**GO ID**	**Description**	**Total genes**	**Partner genes**	***p*-value**
9891	Positive regulation of biosynthetic process	55	12	1.83E-06
31324	Negative regulation of cellular metabolic process	55	12	3.88E-06
9892	Negative regulation of metabolic process	55	12	1.02E-05
44260	Cellular macromolecule metabolic process	55	25	1.33E-05
15248	Sterol transporter activity	20	2	6.56E-05
5159	Insulin-like growth factor receptor binding	24	2	1.57E-04
46627	Negative regulation of insulin receptor signaling pathway	24	2	2.34E-04
33344	Cholesterol efflux	20	2	2.49E-04
30226	Apolipoprotein receptor activity	8	1	4.50E-04
8286	Insulin receptor signaling pathway	20	2	7.39E-04
30301	Cholesterol transport	20	2	8.68E-04
42632	Cholesterol homeostasis	20	2	1.10E-03
55092	Sterol homeostasis	20	2	1.10E-03
34188	Apolipoprotein A-I receptor activity	20	1	1.12E-03
6629	Lipid metabolic process	20	5	1.93E-03
55088	Lipid homeostasis	20	2	2.18E-03
5319	Lipid transporter activity	20	2	2.32E-03
10887	Negative regulation of cholesterol storage	8	1	2.70E-03
5899	Insulin receptor complex	24	1	2.70E-03
32869	Cellular response to insulin stimulus	20	2	3.17E-03
34186	Apolipoprotein A-I binding	20	1	3.37E-03
10875	Positive regulation of cholesterol efflux	8	1	3.59E-03
32365	Intracellular lipid transport	8	1	3.59E-03
10874	Regulation of cholesterol efflux	8	1	4.04E-03
32373	Positive regulation of sterol transport	8	1	4.04E-03
32376	Positive regulation of cholesterol transport	8	1	4.04E-03
34204	Lipid translocation	20	1	4.49E-03
10885	Regulation of cholesterol storage	8	1	5.38E-03
34380	High-density lipoprotein particle assembly	20	1	5.61E-03
43559	Insulin binding	20	1	5.61E-03
10888	Negative regulation of lipid storage	8	1	6.28E-03
70325	Lipoprotein receptor binding	8	1	7.17E-03
31325	Positive regulation of cellular metabolic process	8	3	7.27E-03
32868	Response to insulin stimulus	20	2	8.31E-03
9893	Positive regulation of metabolic process	8	3	8.42E-03
32370	Positive regulation of lipid transport	8	1	8.51E-03
6869	Lipid transport	20	2	1.05E-02
34185	Apolipoprotein binding	20	1	1.23E-02
10876	Lipid localization	20	2	1.23E-02
16071	mRNA metabolic process	24	3	1.30E-02
32368	Regulation of lipid transport	8	1	1.74E-02
9892	Negative regulation of metabolic process	24	4	2.70E-02

Taken together, integrated results of CyTargetLinker target genes analyzed by BiNGO GO terms, suggested that the targeted genes are associated with lipid, cholesterol, lipoprotein, fatty acid, and insulin that significantly involved in their biological, metabolic, and cellular processes. It could be elucidated for their respective pathogenic role and molecular mechanism of action in lipid, cholesterol, lipoprotein, fatty acid, and insulin disorders. In addition, the BiNGO results also highlighted GO terms in cell cycle, cell differentiation, apoptosis/cell death, signaling pathways, protein and carbohydrate metabolisms, immune system, and neuronal metabolism. Therefore, annotated GO terms could help in examining the relationships between the miRNAs and their targets in cancers, metabolic diseases of carbohydrate and proteins, immune diseases, and neurological diseases.

### Performance evaluation

We further compared our study with three other existing databases. For this purpose, we manually checked, confirmed and compared Top 20 miRNAs (of present study) with existing databases such as miR2Disease, miRiaD, and HMDD. We have found that most of the associations missed in miR2Disease and HMDD databases, while miRiaD database missed only few associations shown in Supplementary Table 2. However, Supplementary Figure 1 shows the comparison of our study with miRiaD database. The failure of association in other databases might be due to these databases present most of the miRNAs associations with cancers, while few miRNAs associated with metabolic and other diseases.

## Discussion

The present study is applicable to signify associations between miRNAs and common lipid diseases, where the significant associations were used to visualize and construct the RegIN. The text-mining approach is helpful for extracting the information from huge literature to small subset of extracted information, which is then used for potential knowledge discovery. Hence, we may call the subset information as “literature verified” information.

We are first to provide independently the miRNA-lipid disease associations with network visualization, extension of network for predicted/validated target genes with their associated GO terms. Present study possesses limited number of publication abstracts, although we retrieved abstracts by January 1, 2000 to December 31, 2013. By processing 730 abstracts, we found 227 pairs of miRNA-lipid disease associations, and prioritized 148 miRNAs in nine disease/identifier groups. The major reasons for limited numbers of publications are (a) failure of experimental studies for the discovery and identification of miRNA genes and their targets (Grosswendt et al., 2014), (b) expensive experimental methods for identifying disease related miRNAs and shown low sensitivity & specificity (Jiang et al., 2010, 2013). The co-occurrence based text-mining approach adopted in other studies like, Naeem et al. (2010) for identifying miRNA and genes co-occurring in abstracts; Lu et al. (2008) for identifying miRNA-disease associations; and Jiang et al. (2009) for identifying miRNA-disease relationships. Although, their approaches were effective on limited or low number of publications (100 by Lu et al., 2008 and 600 by Jiang et al., 2009), but not for high scale of text-mining as the number of miRNA research publications increases regularly. In addition, high false positive rate found in their studies, which may lead to poor resolution of miRNA-targets.

We found higher strength of miRNAs in HDL-Cholesterol and Triglyceride groups with higher number of abstracts co-occurring miRNA-33 family. The expression of intronic miRNA-33 family (miR-33a and miR-33b) are from the *sterol regulatory element-binding protein* (*SREBP*) transcription factors, which are known to be involved in cholesterol/lipid homeostasis, and many cholesterogenic/lipogenic genes like *LDL-Receptor, 3-hydroxy-3-methylglutaryl coenzyme A reductase (HMGCR), fatty acid synthase (FAS)* (Brown and Goldstein, 1997, 2009; Horton et al., 2002; Osborne and Espenshade, 2009). The conserved target for miR-33a and miR-33b is *adenosine triphosphate binding cassette A1 (ABCA1)* cholesterol transporter. *ABCA1* helps to transport intracellular cholesterol from liver to apolipoprotein A-1 (apo-A-1) for the synthesis of HDL-C (Maxfield and Tabas, 2005; Wang and Rader, 2007; Tall et al., 2008). At the same extent, by reverse cholesterol transport (RCT) pathway, HDL-C transfers from peripheral tissues/macrophages back to the liver for processing and excretion into bile and feces (Rader et al., 2009). Both increased TGs and decreased HDL-C levels are the characteristics of dyslipidemia, found in insulin-resistant subjects, while the low HDL-C and high TGs level are the hallmarks of atherogenic dyslipidemia both in diabetic and non-diabetic populations (Group, 1997; Jeppesen et al., 1997; Hermans et al., 2012).

By analyzing the top 20 significant miRNAs for the prediction of predicted and validated target genes by CyTargetLinker on Cytoscape, we found the higher number of defined targeted genes as shown in Figure 7. The higher to lower number of targeted genes by miRNAs listed in Table 5 such as, miRNA-155 targeted 31 genes, miRNA-103 targeted 11 genes, and miRNA-200c targeted 10 genes.

Further, targeted genes analyzed by BiNGO for GO annotation, we found more than 90 related GO terms listed in Supplementary Material, among which more than 20 GO terms were significantly associated with lipids, cholesterol, fatty acid, apolipoproteins, sterol, and insulin listed in Table 6. Taking together the defined number of targeted genes by CyTargetLinker with GO terms, suggested that validated and predicted target genes could be regulated *in vivo* by these significant miRNAs in lipid, cholesterol and fatty acid metabolism and associated metabolic diseases. In addition, the miRNAs may be regulated target genes in other non-lipid disorders specially cancers, neurodegenerative disorders, metabolic disorders; and several biological, cellular, and molecular impaired functions. Therefore, for future studies annotated GO terms could help in examining the relationships between the miRNAs and their targets in cancers, metabolic diseases of carbohydrate and proteins, immune diseases and neurological diseases.

## Limitations

There are certain limitations in our study as follows:

1. Limited number of publications in PubMed and Scopus databases as well as duplicate publications. The limited numbers of publications in both databases are due to the limited number of experimental work on disease-related miRNAs owing to the inherent expensive cost and time-consuming nature of the work. As the data in Scopus is limited to only work after 1995, therefore, searching literature from PubMed is preferable to Scopus.
2. High false positive rate is found during text mining. Because, most of the abstracts were mentioned the keyword microRNA/miRNA but not mentioned lipid disease/identifier names. Therefore, abstracts should contains information of both miRNA and disease name.

## Conclusion

To the best of our knowledge, this study represents the first study to provide reliable miRNA-lipid disease association network based on text-mining method. We extracted 227 miRNA-lipid disease associations between 148 miRNAs and nine common lipid diseases/identifiers from bulk published data. In the present study significant groups such as, HDL-C, dyslipidemia and triglyceride should be evaluated further for identifying the complex involvement of miRNAs and disease development. We also constructed extended RegIN from top 20 significant text-mined miRNAs using CyTargetLinker on Cytoscape that provides experimentally validated and predicted miRNA gene targets. Further, these miRNA gene targets are involved in the regulation of lipid, cholesterol, lipoprotein, and fatty acid biological processes, which are confirmed by BiNGO analysis on Cytoscape.

The current study sets the groundwork for future experimental studies to validate the targeted mRNAs/genes, since they have been predicted with CyTargetLinker but not experimentally validated. Future experimental studies could walk around the biological functions and primary molecular mechanism of miRNAs in the development, progression, diagnosis and prognosis of lipid and cholesterol, lipoprotein, and fatty acid disorders.

## Author contributions

AK is a Ph. D. student and made substantial contributions in analyzing and interpreting the research results as well as manuscript preparation. WS is a co-advisor and verified the analysis and guidance specifically in statistics. PN is a major advisor and participated in study design, preparation and reviewing of the drafted manuscript. CN and VP gave a critical review of the manuscript.

### Conflict of interest statement

The authors declare that the research was conducted in the absence of any commercial or financial relationships that could be construed as a potential conflict of interest.
