# Supplementary material for: The MicroRNA Interaction Network of Lipid Diseases
Source: Front Genet. 2017 Sep 22;8:116. doi: 10.3389/fgene.2017.00116 (PMC5615414; doi:10.3389/fgene.2017.00116)
Supplement: Supplementary file 1 [file Supplementarymaterial.doc]

**“Supplementary material”**

**Supplementary Table 1** Gene Ontology terms of CyTargetLinker targeted genes analysed by BINGO on Cytoscape

| **GO ID** | **Description** | **Total genes** | **Partner genes** | **pValue** | **Significant /**  **Non-Sig. Go terms** |
| --- | --- | --- | --- | --- | --- |
| **48519** | negative regulation of biological process | 55 | 22 | 4.53E-08 | Significant GO terms |
| **50789** | regulation of biological process | 55 | 40 | 6.56E-08 | Significant GO terms |
| **65007** | biological regulation | 55 | 41 | 9.11E-08 | Significant GO terms |
| **9891** | positive regulation of biosynthetic process | 55 | 12 | 1.83E-06 | Significant GO terms |
| **31324** | negative regulation of cellular metabolic process | 55 | 12 | 3.88E-06 | Significant GO terms |
| **9892** | negative regulation of metabolic process | 55 | 12 | 1.02E-05 | Significant GO terms |
| **44260** | cellular macromolecule metabolic process | 55 | 25 | 1.33E-05 | Significant GO terms |
| **9890** | negative regulation of biosynthetic process | 55 | 10 | 1.77E-05 | Significant GO terms |
| **48518** | positive regulation of biological process | 55 | 19 | 1.86E-05 | Significant GO terms |
| **44424** | intracellular part | 55 | 48 | 2.45E-05 | Significant GO terms |
| **43231** | intracellular membrane-bounded organelle | 55 | 41 | 2.83E-05 | Significant GO terms |
| **43170** | macromolecule metabolic process | 55 | 26 | 4.67E-05 | Significant GO terms |
| **48519** | negative regulation of biological process | 8 | 6 | 4.86E-05 | Significant GO terms |
| **15248** | sterol transporter activity | 20 | 2 | 6.56E-05 | Significant GO terms |
| **5622** | intracellular | 55 | 48 | 7.99E-05 | Significant GO terms |
| **5488** | binding | 55 | 50 | 1.45E-04 | Significant GO terms |
| **48519** | negative regulation of biological process | 24 | 10 | 1.52E-04 | Significant GO terms |
| **5159** | insulin-like growth factor receptor binding | 24 | 2 | 1.57E-04 | Significant GO terms |
| **65008** | regulation of biological quality | 8 | 5 | 2.18E-04 | Significant GO terms |
| **46627** | negative regulation of insulin receptor signaling pathway | 24 | 2 | 2.34E-04 | Significant GO terms |
| **33344** | cholesterol efflux | 20 | 2 | 2.49E-04 | Significant GO terms |
| **46626** | regulation of insulin receptor signaling pathway | 24 | 2 | 3.61E-04 | Significant GO terms |
| **30226** | apolipoprotein receptor activity | 8 | 1 | 4.50E-04 | Significant GO terms |
| **34188** | apolipoprotein A-I receptor activity | 8 | 1 | 4.50E-04 | Significant GO terms |
| **8286** | insulin receptor signaling pathway | 20 | 2 | 7.39E-04 | Significant GO terms |
| **44237** | cellular metabolic process | 55 | 27 | 7.40E-04 | Significant GO terms |
| **50789** | regulation of biological process | 24 | 17 | 7.45E-04 | Significant GO terms |
| **15918** | sterol transport | 20 | 2 | 8.68E-04 | Significant GO terms |
| **30301** | cholesterol transport | 20 | 2 | 8.68E-04 | Significant GO terms |
| **48519** | negative regulation of biological process | 20 | 8 | 9.71E-04 | Significant GO terms |
| **42632** | cholesterol homeostasis | 20 | 2 | 1.10E-03 | Significant GO terms |
| **55092** | sterol homeostasis | 20 | 2 | 1.10E-03 | Significant GO terms |
| **30226** | apolipoprotein receptor activity | 20 | 1 | 1.12E-03 | Significant GO terms |
| **34188** | apolipoprotein A-I receptor activity | 20 | 1 | 1.12E-03 | Significant GO terms |
| **34186** | apolipoprotein A-I binding | 8 | 1 | 1.35E-03 | Significant GO terms |
| **8202** | steroid metabolic process | 20 | 3 | 1.55E-03 | Significant GO terms |
| **65007** | biological regulation | 24 | 17 | 1.59E-03 | Significant GO terms |
| **31323** | regulation of cellular metabolic process | 8 | 6 | 1.61E-03 | Significant GO terms |
| **34204** | lipid translocation | 8 | 1 | 1.80E-03 | Significant GO terms |
| **5488** | binding | 24 | 23 | 1.86E-03 | Significant GO terms |
| **6629** | lipid metabolic process | 20 | 5 | 1.93E-03 | Significant GO terms |
| **19222** | regulation of metabolic process | 8 | 6 | 2.10E-03 | Significant GO terms |
| **55088** | lipid homeostasis | 20 | 2 | 2.18E-03 | Significant GO terms |
| **32367** | intracellular cholesterol transport | 8 | 1 | 2.25E-03 | Significant GO terms |
| **5319** | lipid transporter activity | 20 | 2 | 2.32E-03 | Significant GO terms |
| **9987** | cellular process | 20 | 17 | 2.68E-03 | Significant GO terms |
| **10887** | negative regulation of cholesterol storage | 8 | 1 | 2.70E-03 | Significant GO terms |
| **5899** | insulin receptor complex | 24 | 1 | 2.70E-03 | Significant GO terms |
| **8150** | biological_process | 55 | 52 | 2.96E-03 | Significant GO terms |
| **32869** | cellular response to insulin stimulus | 20 | 2 | 3.17E-03 | Significant GO terms |
| **48545** | response to steroid hormone stimulus | 24 | 3 | 3.32E-03 | Significant GO terms |
| **44260** | cellular macromolecule metabolic process | 24 | 11 | 3.35E-03 | Significant GO terms |
| **34186** | apolipoprotein A-I binding | 20 | 1 | 3.37E-03 | Significant GO terms |
| **10875** | positive regulation of cholesterol efflux | 8 | 1 | 3.59E-03 | Significant GO terms |
| **32365** | intracellular lipid transport | 8 | 1 | 3.59E-03 | Significant GO terms |
| **44444** | cytoplasmic part | 20 | 12 | 3.69E-03 | Significant GO terms |
| **10874** | regulation of cholesterol efflux | 8 | 1 | 4.04E-03 | Significant GO terms |
| **32373** | positive regulation of sterol transport | 8 | 1 | 4.04E-03 | Significant GO terms |
| **32376** | positive regulation of cholesterol transport | 8 | 1 | 4.04E-03 | Significant GO terms |
| **5010** | insulin-like growth factor receptor activity | 20 | 1 | 4.49E-03 | Significant GO terms |
| **34204** | lipid translocation | 20 | 1 | 4.49E-03 | Significant GO terms |
| **15248** | sterol transporter activity | 8 | 1 | 4.94E-03 | Significant GO terms |
| **34185** | apolipoprotein binding | 8 | 1 | 4.94E-03 | Significant GO terms |
| **50789** | regulation of biological process | 8 | 7 | 4.98E-03 | Significant GO terms |
| **8150** | biological_process | 24 | 24 | 5.32E-03 | Significant GO terms |
| **10885** | regulation of cholesterol storage | 8 | 1 | 5.38E-03 | Significant GO terms |
| **5010** | insulin-like growth factor receptor activity | 24 | 1 | 5.39E-03 | Significant GO terms |
| **32366** | intracellular sterol transport | 20 | 1 | 5.61E-03 | Significant GO terms |
| **32367** | intracellular cholesterol transport | 20 | 1 | 5.61E-03 | Significant GO terms |
| **34380** | high-density lipoprotein particle assembly | 20 | 1 | 5.61E-03 | Significant GO terms |
| **43559** | insulin binding | 20 | 1 | 5.61E-03 | Significant GO terms |
| **10888** | negative regulation of lipid storage | 8 | 1 | 6.28E-03 | Significant GO terms |
| **10887** | negative regulation of cholesterol storage | 20 | 1 | 6.73E-03 | Significant GO terms |
| **31994** | insulin-like growth factor I binding | 20 | 1 | 6.73E-03 | Significant GO terms |
| **70325** | lipoprotein receptor binding | 8 | 1 | 7.17E-03 | Significant GO terms |
| **65007** | biological regulation | 8 | 7 | 7.25E-03 | Significant GO terms |
| **31325** | positive regulation of cellular metabolic process | 8 | 3 | 7.27E-03 | Significant GO terms |
| **48522** | positive regulation of cellular process | 8 | 4 | 7.73E-03 | Significant GO terms |
| **32868** | response to insulin stimulus | 20 | 2 | 8.31E-03 | Significant GO terms |
| **9893** | positive regulation of metabolic process | 8 | 3 | 8.42E-03 | Significant GO terms |
| **32370** | positive regulation of lipid transport | 8 | 1 | 8.51E-03 | Significant GO terms |
| **34645** | cellular macromolecule biosynthetic process | 8 | 3 | 8.77E-03 | Significant GO terms |
| **32365** | intracellular lipid transport | 20 | 1 | 8.96E-03 | Significant GO terms |
| **9059** | macromolecule biosynthetic process | 8 | 3 | 9.27E-03 | Significant GO terms |
| **65009** | regulation of molecular function | 8 | 3 | 9.47E-03 | Significant GO terms |
| **44260** | cellular macromolecule metabolic process | 8 | 5 | 9.76E-03 | Significant GO terms |
| **50789** | regulation of biological process | 20 | 13 | 9.79E-03 | Significant GO terms |
| **43170** | macromolecule metabolic process | 24 | 11 | 9.88E-03 | Significant GO terms |
| **44237** | cellular metabolic process | 20 | 11 | 1.01E-02 | Significant GO terms |
| **32371** | regulation of sterol transport | 8 | 1 | 1.03E-02 | Significant GO terms |
| **32374** | regulation of cholesterol transport | 8 | 1 | 1.03E-02 | Significant GO terms |
| **6869** | lipid transport | 20 | 2 | 1.05E-02 | Significant GO terms |
| **43550** | regulation of lipid kinase activity | 24 | 1 | 1.07E-02 | Significant GO terms |
| **32934** | sterol binding | 8 | 1 | 1.12E-02 | Significant GO terms |
| **34185** | apolipoprotein binding | 20 | 1 | 1.23E-02 | Significant GO terms |
| **10876** | lipid localization | 20 | 2 | 1.23E-02 | Significant GO terms |
| **5010** | insulin-like growth factor receptor activity | 55 | 1 | 1.23E-02 | Significant GO terms |
| **16071** | mRNA metabolic process | 24 | 3 | 1.30E-02 | Significant GO terms |
| **10885** | regulation of cholesterol storage | 20 | 1 | 1.34E-02 | Significant GO terms |
| **5737** | cytoplasm | 20 | 14 | 1.36E-02 | Significant GO terms |
| **10888** | negative regulation of lipid storage | 20 | 1 | 1.56E-02 | Significant GO terms |
| **44238** | primary metabolic process | 20 | 11 | 1.59E-02 | Significant GO terms |
| **65007** | biological regulation | 20 | 13 | 1.68E-02 | Significant GO terms |
| **15918** | sterol transport | 8 | 1 | 1.74E-02 | Significant GO terms |
| **32368** | regulation of lipid transport | 8 | 1 | 1.74E-02 | Significant GO terms |
| **43170** | macromolecule metabolic process | 8 | 5 | 1.76E-02 | Significant GO terms |
| **44237** | cellular metabolic process | 24 | 12 | 1.85E-02 | Significant GO terms |
| **31325** | positive regulation of cellular metabolic process | 20 | 4 | 2.09E-02 | Significant GO terms |
| **9893** | positive regulation of metabolic process | 20 | 4 | 2.48E-02 | Significant GO terms |
| **6706** | steroid catabolic process | 20 | 1 | 2.56E-02 | Significant GO terms |
| **5520** | insulin-like growth factor binding | 20 | 1 | 2.67E-02 | Significant GO terms |
| **9892** | negative regulation of metabolic process | 24 | 4 | 2.70E-02 | Significant GO terms |
| **55088** | lipid homeostasis | 8 | 1 | 2.75E-02 | Significant GO terms |
| **5319** | lipid transporter activity | 8 | 1 | 2.84E-02 | Significant GO terms |
| **5496** | steroid binding | 8 | 1 | 2.93E-02 | Significant GO terms |
| **48545** | response to steroid hormone stimulus | 55 | 3 | 3.24E-02 | Significant GO terms |
| **9889** | regulation of biosynthetic process | 8 | 4 | 3.35E-02 | Significant GO terms |
| **5622** | intracellular | 20 | 17 | 3.35E-02 | Significant GO terms |
| **8152** | metabolic process | 20 | 11 | 3.89E-02 | Significant GO terms |
| **44237** | cellular metabolic process | 8 | 5 | 4.41E-02 | Significant GO terms |
| **31323** | regulation of cellular metabolic process | 24 | 9 | 4.78E-02 | Significant GO terms |
| **35258** | steroid hormone receptor binding | 20 | 1 | 4.94E-02 | Significant GO terms |
| **3674** | molecular_function | 20 | 20 | 5.89E-02 | Non Significant GO terms |
| **5623** | cell | 55 | 51 | 5.94E-02 | Non Significant GO terms |
| **6869** | lipid transport | 8 | 1 | 6.08E-02 | Non Significant GO terms |
| **10876** | lipid localization | 8 | 1 | 6.59E-02 | Non Significant GO terms |
| **8152** | metabolic process | 24 | 12 | 6.98E-02 | Non Significant GO terms |
| **44260** | cellular macromolecule metabolic process | 20 | 7 | 8.12E-02 | Non Significant GO terms |
| **8152** | metabolic process | 8 | 5 | 8.96E-02 | Non Significant GO terms |
| **44092** | negative regulation of molecular function | 24 | 2 | 8.98E-02 | Non Significant GO terms |
| **48518** | positive regulation of biological process | 20 | 5 | 9.27E-02 | Non Significant GO terms |
| **31323** | regulation of cellular metabolic process | 20 | 7 | 1.07E-01 | Non Significant GO terms |
| **43170** | macromolecule metabolic process | 20 | 7 | 1.44E-01 | Non Significant GO terms |
| **44424** | intracellular part | 20 | 15 | 1.57E-01 | Non Significant GO terms |
| **44464** | cell part | 20 | 19 | 1.63E-01 | Non Significant GO terms |
| **5623** | cell | 20 | 19 | 1.63E-01 | Non Significant GO terms |
| **19216** | regulation of lipid metabolic process | 24 | 1 | 1.65E-01 | Non Significant GO terms |
| **8289** | lipid binding | 8 | 1 | 1.71E-01 | Non Significant GO terms |
| **16042** | lipid catabolic process | 20 | 1 | 1.85E-01 | Non Significant GO terms |
| **8150** | biological_process | 20 | 18 | 2.19E-01 | Non Significant GO terms |
| **5488** | binding | 20 | 16 | 2.23E-01 | Non Significant GO terms |
| **5488** | binding | 8 | 7 | 2.46E-01 | Non Significant GO terms |
| **5622** | intracellular | 8 | 6 | 3.94E-01 | Non Significant GO terms |
| **44265** | cellular macromolecule catabolic process | 20 | 1 | 3.95E-01 | Non Significant GO terms |
| **8150** | biological_process | 8 | 7 | 5.15E-01 | Non Significant GO terms |
| **5575** | cellular_component | 20 | 19 | 5.21E-01 | Non Significant GO terms |
| **65008** | regulation of biological quality | 24 | 2 | 6.28E-01 | Non Significant GO terms |
| **9892** | negative regulation of metabolic process | 20 | 1 | 6.31E-01 | Non Significant GO terms |
| **44464** | cell part | 8 | 7 | 6.44E-01 | Non Significant GO terms |
| **5623** | cell | 8 | 7 | 6.44E-01 | Non Significant GO terms |
| **34645** | cellular macromolecule biosynthetic process | 24 | 1 | 7.62E-01 | Non Significant GO terms |
| **9059** | macromolecule biosynthetic process | 24 | 1 | 7.69E-01 | Non Significant GO terms |
| **44249** | cellular biosynthetic process | 24 | 1 | 9.09E-01 | Non Significant GO terms |
| **9058** | biosynthetic process | 24 | 1 | 9.22E-01 | Non Significant GO terms |

**Supplementary Table 2** Comparison of Co-occurrence based Text mining work of present study with existing databases

| **Top 20 miRNAs** | **miRiaD** | **HMDD v.2** | **miR2Disease** | **Our Study** |
| --- | --- | --- | --- | --- |
| **miRNA-33a** | 4 | 0 | 0 | **7** |
| **miRNA-33a/b** | 4 | 0 | 0 | **7** |
| **miRNA-144** | 1 | 0 | 0 | **3** |
| **miRNA-223** | 2 | 0 | 0 | **3** |
| **miRNA-33b** | 1 | 0 | 0 | **3** |
| **miRNA-185** | 0 | 0 | 0 | **2** |
| **miRNA-96** | 0 | 0 | 0 | **2** |
| **miRNA-103** | 0 | 0 | 0 | **2** |
| **miRNA-122a** | 0 | 0 | 0 | **2** |
| **miRNA-17-92** | 1 | 0 | 0 | **2** |
| **miRNA-375** | 1 | 0 | 0 | **2** |
| **miRNA-122** | 2 | 3 | 0 | **5** |
| **miRNA-33** | 4 | 0 | 0 | **16** |
| **miRNA-200c** | 0 | 0 | 0 | **2** |
| **miRNA-126** | 1 | 1 | 0 | **2** |
| **miRNA-30c** | 0 | 0 | 0 | **2** |
| **miRNA-145** | 0 | 0 | 0 | **2** |
| **miRNA-29b** | 0 | 0 | 0 | **3** |
| **miRNA-155** | 3 | 0 | 0 | **2** |
| **miRNA-146a** | 1 | 0 | 0 | **4** |

**
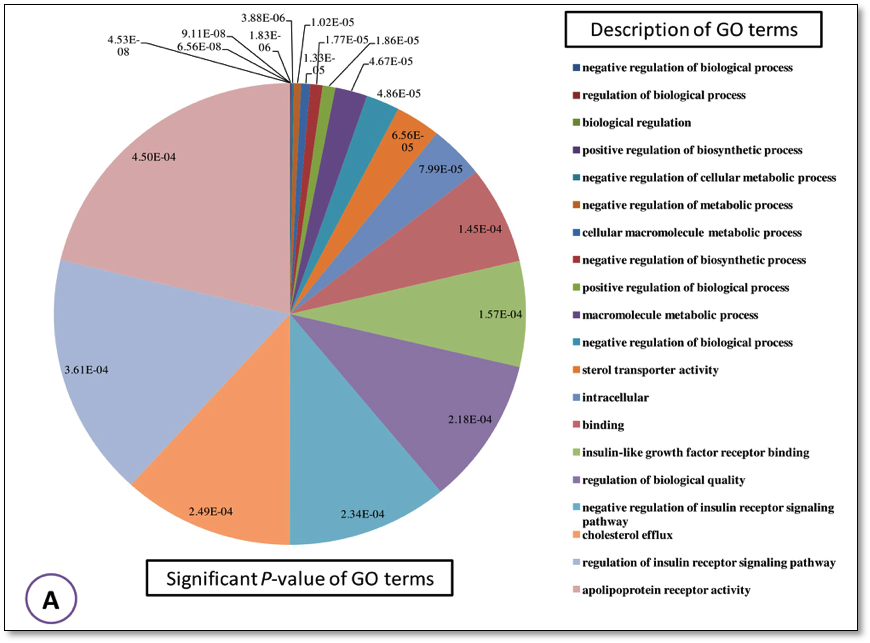
**


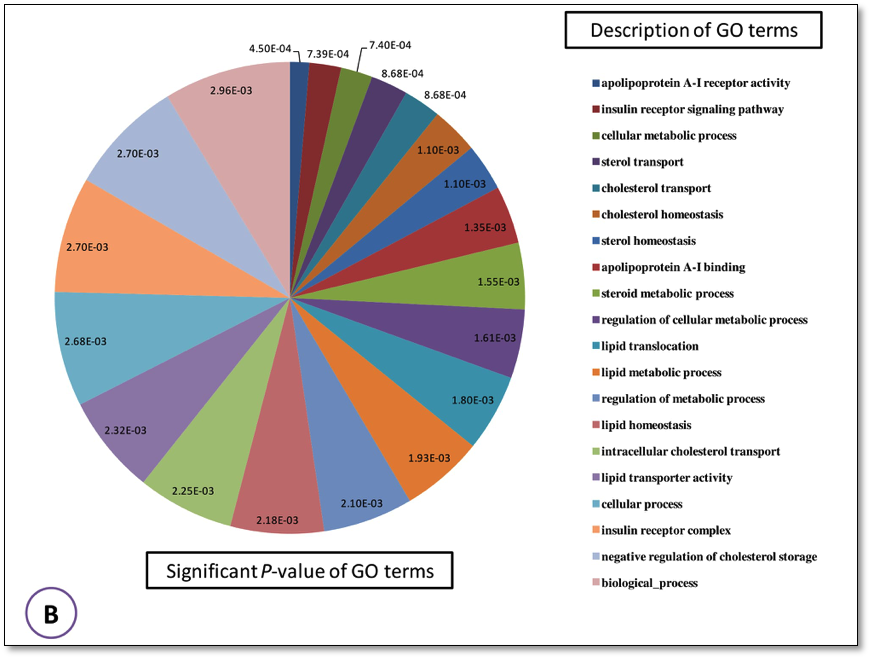


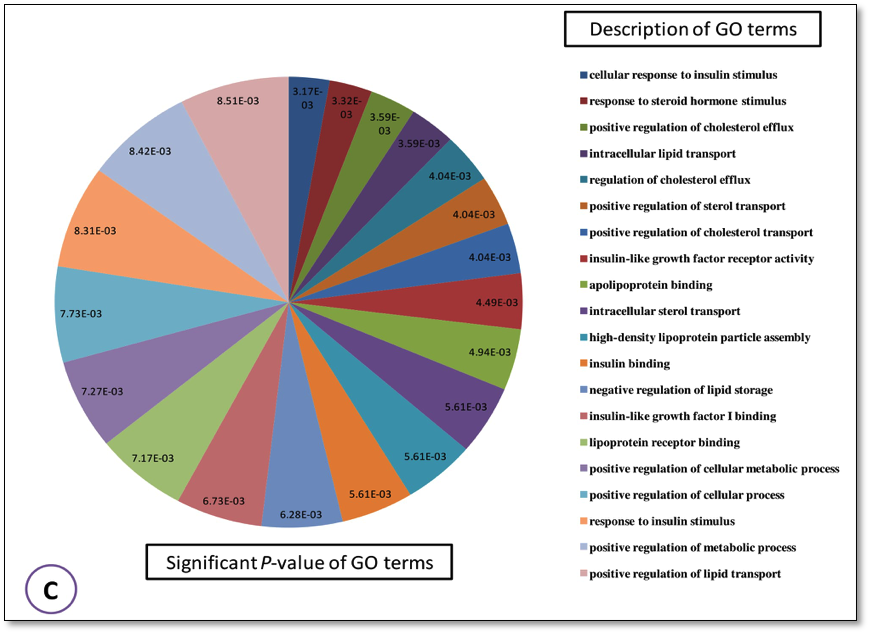


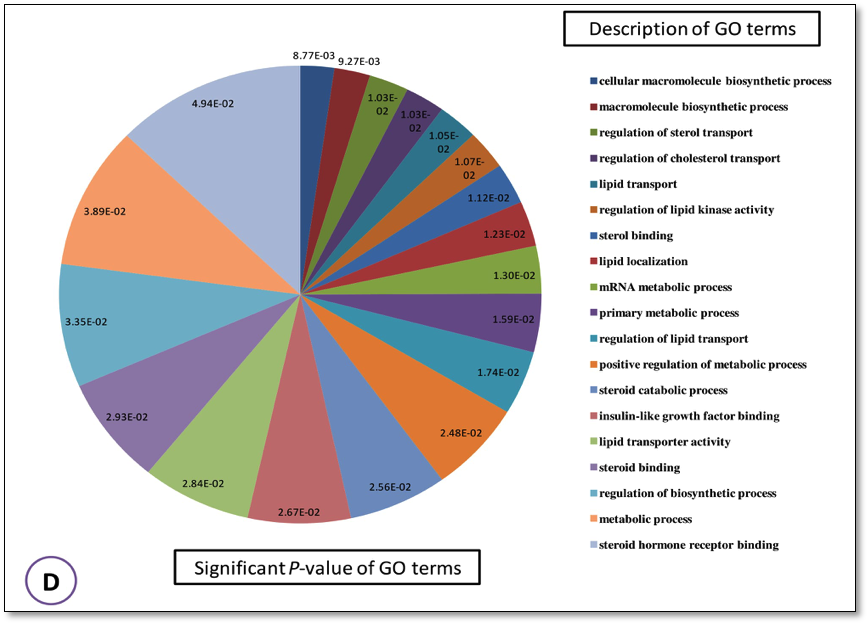


**Supplementary Figure 1 (A, B, C, and D):** Significant Gene ontology terms including lipids, cholesterol, and fatty acid biological processes.

Significant GO terms derived from Supplementary Table 1, Pie charts labeled with P-values, while colored boxes with description of the GO terms panel at the right side.


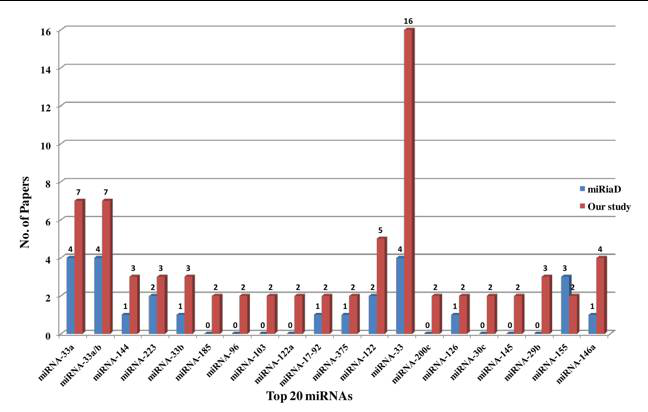


**Supplementary figure 2** Comparioson of top 20 miRNAs associations among miRiaD online database and current study. Red bars shows present study and blue bars shows miRiaD database, each bar represents numbers corresponding number of papers for particular miRNAs.
